# Fecal Calprotectin, Elastase, and Alpha-1-Antitrypsin Levels After Roux-en-Y Gastric Bypass; Calprotectin Is Significantly Elevated in the Majority of Patients

**DOI:** 10.1007/s11695-016-2222-0

**Published:** 2016-05-23

**Authors:** Thomas C. C. Boerlage, Floris Westerink, Dennis C. W. Poland, Inge L. Huibregtse, Yair I. Z. Acherman, Victor E. A. Gerdes

**Affiliations:** MC Slotervaart, Louwesweg 6, Amsterdam, 1066EC The Netherlands

**Keywords:** Fecal tests, Calprotectin, Pancreatic elastase, Alpha-1-antitrypsin, Roux-en-Y gastric bypass

## Abstract

**Background:**

Roux-en-Y gastric bypass (RYGB) causes several alterations in gastrointestinal function. We hypothesized that levels of three commonly used fecal tests change after RYGB.

**Methods:**

Fecal levels of calprotectin, elastase, and alpha-1-antitrypsin were determined in 122 patients without signs of gastrointestinal disease 1 to 2 years after RYGB. Medians, distribution of values, and the percentage of patients with levels above or below reference values were determined.

**Results:**

Median fecal calprotectin level was 163.5 (<30–1587) μg/g; in 87 % of patients, it was above the reference value (<50 μg/g). Median fecal elastase level was 444 (<15–647) μg/g; 13 % was below the reference value (>200 μg/g). Median fecal alpha-1-antitrypsin level was 0.51 (<0.20–2.20) mg/g, comparable to the reference values.

**Conclusions:**

Fecal calprotectin levels are significantly higher than the reference value in most patients after RYGB. Fecal elastase is significantly lower. This might indicate that the validity of fecal calprotectin testing is impaired after RYGB and the specificity for fecal elastase is decreased. Clinical awareness of altered fecal markers after RYGB is essential to prevent unnecessary diagnostic tests, such as colonoscopy. Fecal alpha-1-antitrypsin is not influenced by RYGB.

## Introduction

Bariatric surgery is the most effective treatment for morbid obesity in the long term. Roux-en-Y gastric bypass (RYGB) is most commonly performed worldwide [1]. RYGB has a restrictive effect on food intake and results in delayed mixing of the food with bile and pancreatic enzymes. RYGB is also known to alter the excretion and plasma concentration of several hormones, the fecal bacterial flora, the absorption of several drugs, and patients’ bowel habits [2–5]. Taking these changes in gastrointestinal function after RYGB in account, we hypothesized that the level of fecal markers might also change. This is relevant for several fecal proteins and enzymes that are used to screen for gastrointestinal diseases or for follow-up of disease severity. Three commonly used fecal tests are calprotectin, pancreatic elastase-1 (elastase), and alpha-1-antitrypsin (A1AT). A recent article showed significant differences in fecal levels of calprotectin and elastase between seven patients after RYGB and obese controls [6].

Calprotectin is a protein present in most cells, but especially in the neutrophil granulocyte, where it makes up 60 % of the cytoplasmic proteins. Neutrophils in the intestinal wall secrete calprotectin in case of intestinal inflammation. It is resistant against proteolysis, and after secretion, it is not reabsorbed or metabolized in the intestine [7]. Therefore, it is excreted in the feces and can be used to differentiate between inflammatory bowel disease (IBD) and functional complaints with high sensitivity and specificity. It is also used for IBD follow-up [8].

Elastase-1 is a pancreas-specific enzyme secreted by the pancreas for digestion that remains stable and is not reabsorbed throughout the intestinal tract [9]. Determination of elastase in the feces is a cheap, reliable, and non-invasive test and therefore generally used for diagnosing exocrine pancreatic insufficiency [10]. Secretion of pancreatic juices and bile is regulated by several different gut hormones. Those are, in turn, secreted in response to alterations in duodenal food content and acidity [11]. Due to the altered anatomy after RYGB, plasma concentration of these gut hormones changes. This can result in decreased secretion of pancreatic enzymes such as elastase [6, 11].

Alpha-1-antitrypsin is a protein produced in the liver with immunomodulatory effects, mainly through inhibition of neutrophil elastase. It is excreted in the feces in small amounts, is not metabolized or reabsorbed in the intestine, and remains stable in the feces when stored [12]. Before the implementation of fecal calprotectin tests, A1AT was used in the diagnosis and follow-up of IBD. Nowadays, it is mainly used as a marker for intestinal leakage of plasma proteins, as happens in protein-losing enteropathy [13].

This study was designed to determine fecal levels of calprotectin, pancreatic elastase-1, and alpha-1-antitrypsin after RYGB. We hypothesized that the fecal levels of these proteins change significantly after surgery.

## Methods

We performed an observational cross-sectional study to determine fecal values in patients after Roux-en-Y gastric bypass. We compared these levels with the general population and with the current reference values. The reference values for calprotectin and elastase are not based on healthy reference interval calculation, but are medical decision limits, based on the predictive value for underlying disease. Therefore, it is not possible to calculate a new reference value for calprotectin and elastase with this study design. The reference value for A1AT is based on healthy population intervals, so it is possible to calculate a new reference value for A1AT [12].

Consecutive patients visiting the outpatient clinic for follow-up 1 to 2 years after RYGB were asked to participate. This time period was chosen to make sure patients were stable in weight and gastrointestinal symptoms, and the jejunal mucosa had adapted to the new situation [14]. All patients fulfilled the criteria for bariatric surgery of a body mass index (BMI) of >40 kg/m^2^ or >35 kg/m^2^ with comorbidity before surgery. All patients had a laparoscopic antegastric antecolic RYGB with a biliopancreatic limb of 50 cm.

Exclusion criteria were any acute or chronic gastrointestinal disease or systemic disease involving the intestine. This included IBD, gastrointestinal malignancy, chronic pancreatitis, intestinal infection, bowel resection, radiotherapy involving the gastrointestinal area, or watery stools at time of collection. A history of cholecystectomy, acute pancreatitis, gastro-enteritis, or any other acute gastrointestinal disease was not an exclusion criterion if the patient had fully recovered. It is reported that use of a proton pump inhibitor (PPI) or non-steroidal anti-inflammatory drug (NSAID) might influence fecal calprotectin levels [15, 16]. Patients using a PPI or NSAID discontinued this at least 3 days prior to feces collection. If discontinuation was not possible, patients were excluded.

The feces was collected by the patient at home using the feces collection device Fe-Col® (Greiner Bio-One, Kremsmünster, Austria) and delivered to the hospital within 24 h. It was stored at −20 °C, and all specimens were defrosted and analyzed in one batch by the laboratory for clinical chemistry and hematology of the University Medical Center Utrecht.

The medical ethics committee of the Slotervaart Hospital/Reade in Amsterdam gave approval for this study (NL49803.048.14, P1437). All patients gave written informed consent. The study was registered in the Netherlands Trial Register (www.trialregister.nl, number 4761).

### Laboratory Methods

Calprotectin was analyzed by enzyme-linked immunosorbent assay (ELISA) after preparation (Bühlmann Laboratories AG, Schönenbuch, Switzerland). Results are presented in microgram calprotectin per gram feces (μg/g). The reference value for this test is <50 μg/g, with a lower detection limit of 30 μg/g. The median level in the population ranges from 9.3 to 31 μg/g, with all larger studies showing a median calprotectin level below 30 μg/g [8]. The median level in obese people is comparable to the general population [17]. A median of 30 μg/g in the general population was assumed for comparison with the results of this study.

Elastase was analyzed using ELISA (ScheBo Biotec AG, Giessen, Germany). Results are presented in microgram per gram. The lower limit for reliable determination is 15 μg/g; the upper limit is 500 μg/g. In case of a level of >500 μg/g, the exact level was also determined but is known to be less reliable. An elastase level of less than 200 μg/g indicates exocrine pancreatic insufficiency; less than 100 μg/g indicates severe pancreatic insufficiency. The median level in the population is 478 μg/g [10].

For A1AT determination, feces was freeze-dried followed by nephelometry (Siemens Healthcare Diagnostics Products GmbH, Marburg, Germany). Results are presented in milligram A1AT per gram dry feces (mg/g). An A1AT level of more than 1.10 mg/g (54 mg/dL) is considered an indication of gastrointestinal protein loss. The lower detection limit depends on the quality of the sample and can vary between 0.20 and 0.80 mg/g. No median level in the general population is known for A1AT; therefore, the known mean level of 0.58 mg/g was used for comparison [18].

### Statistical Methods

Power was determined according to the Clinical and Laboratory Standards Institute (CLSI) guideline for determination of reference values. When a reference value is determined in a population that significantly differs from the general population, at least 120 persons should be included [19]. Therefore, we aimed to include 120 patients.

Data were analyzed using SPSS Statistics version 21 (IBM Corp. 2012). Median, interquartile range (IQR), and total range are given because of non-normal distribution. For levels that were above or below the limit for reliable detection, the result is presented as this limit.

Subsequently, subgroup analyses of sex, previous cholecystectomy, previous bariatric surgery, all previous abdominal surgery combined, and recent (<7 days) versus longer discontinuation of PPI were performed for all three fecal tests with Mann-Whitney *U* test.

Correlations were determined for the most recent known serum C-reactive protein (CRP) level, age, number of months postoperative, current BMI, and percentage total weight loss for all three fecal tests, using Spearman’s rho test. Furthermore, the mutual correlations between the fecal tests were determined.

Because multiple correlations and subgroup analyses were performed for each test, Bonferroni correction was used. A *p* value of <0.005 was considered statistically significant.

The median fecal levels in this study were compared with the previously published median, or mean in case of A1AT, values in the population using the sign (one-sample binomial) test. A *p* value of <0.05 was considered statistically significant.

The reference value for A1AT was determined in accordance with the CLSI guideline [19]. Outliers were first identified and removed using the method described by Dixon [20]. The 97.5th percentile was considered the reference value. The 90 % confidence interval was identified using the rank method, with the highest rank number and the seventh highest rank number representing the upper and lower limit of the confidence interval [21].

## Results

Between October and December 2014, 122 patients were included. Characteristics of the included patients are shown in Table 1.

**Table 1:** Patient characteristics

	*N* (%)
Sex: female/male	108 (88.5 %)/14 (11.5 %)
PPI discontinued <7 days	79 (64.8 %)
Prior bariatric surgery	12 (9.8 %)
Cholecystectomy	26 (21.3 %)
Total prior abdominal surgery	55 (45.1 %)
	Median (range)
Age (years)	47 (24–64)
Time postoperative (months)	13 (12–24)
Current weight (kilogram)	82.1 (53–134)
Total weight loss (%)	31.6 (5.7–51.0)
CRP serum level (mg/l)	1.1 (0.1–32.8)

Subgroups are presented in number of patients (*N*) and percentages in parentheses (%). Continuous data is expressed as median and range in parentheses

*PPI* proton pump inhibitor, *CRP* C-reactive protein

### Calprotectin

**Fig. 1:**
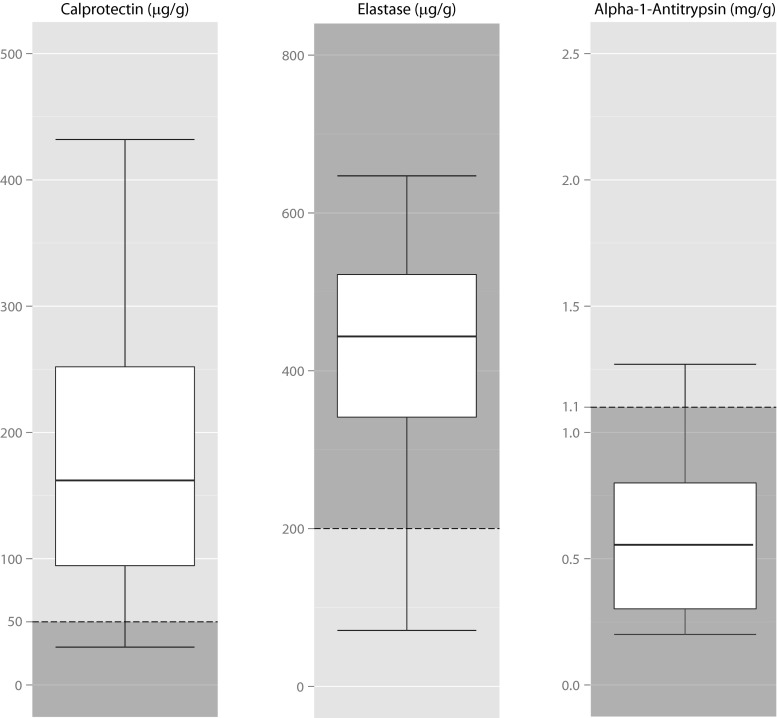
Boxplots of calprotectin, elastase, and alpha-1-antitrypsin. Normal range is presented in *dark*. Outliers (1 for alpha-1-antitrypsin, 3 for calprotectin, 8 for elastase) are not shown

The median calprotectin level was 163.5 μg/g, ranging from <30 μg/g in ten participants to 1587 μg/g. One hundred four patients (87.4 %) had a calprotectin level above the current reference value of 50 μg/g (Table 2; Fig. 1). The median fecal calprotectin level was significantly different from the previously published median level in the general and obese population of 30 μg/g (*p* < 0.001). Subgroup analyses revealed no differences in median calprotectin levels between groups (Table 3). There was no correlation with any of the patient characteristics (Table 4), but there was a significant correlation with the fecal A1AT level (rho = 0.492, *p* < 0.001).

**Table 2:** Results of fecal tests

	Calprotectin (μg/g)	Elastase (μg/g)	Alpha-1-antitrypsin (mg/g)
Number of patients	122	122	115
Median	164	444	0.51
Minimum	<30	<15	<0.20
Percentiles			
25	95	339	0.30
75	257	545	0.80
Maximum	1587	647	2.20
Regular reference value	<50	>200	<1.10

Alpha-1-antitrypsin level could not be determined reliably in seven patients

*μg/g* microgram per gram feces, *mg/g* milligram per gram dry feces

**Table 3:** Subgroup analyses

		Calprotectin (μg/g)		Elastase (μg/g)		Alpha-1-antitrypsin (mg/g)	
		Median (IQR)		Median (IQR)		Median (IQR)	
Sex	Female	185 (97–264)	*p* = 0.15	444 (363–524)	*p* = 0.18	0.55 (0.31–0.80)	*p* = 0.37
	Male	130 (91–190)		362 (249–490)		0.43 (0.24–0.66)	
PPI discontinued	≤7 days	191 (98–270)	*p* = 0.23	443 (341–519)	*p* = 0.95	0.58 (0.34–0.80)	*p* = 0.35
	>7 days	158 (79–237)		444 (332–530)		0.45 (0.23–0.81)	
Cholecystectomy	Yes	193 (135–302)	*p* = 0.12	481 (350–545)	*p* = 0.18	0.60 (0.49–0.80)	*p* = 0.15
	No	162 (89–254)		435 (334–507)		0.48 (0.24–0.81)	
Prior bariatric surgery	Yes	196 (115–304)	*p* = 0.41	515 (398–562)	*p* = 0.16	0.46 (0.31–0.72)	*p* = 0.58
	No	164 (93–256)		443 (333–510)		0.56 (0.30–0.81)	
Any prior abdominal surgery	Yes	208 (89–270)	*p* = 0.54	464 (326–544)	*p* = 0.53	0.57 (0.36–0.80)	*p* = 0.31
	No	147 (106–234)		438 (342–490)		0.48 (0.20–0.81)	

Data expressed in median and interquartile range (IQR). *p* value was calculated with Mann-Whitney *U. p* < 0.005 was considered statistically significant

*PPI* proton pump inhibitor, *μg/g* microgram per gram feces, *mg/g* milligram per gram dry feces

**Table 4:** Correlations with patient characteristics

	Calprotectin	Elastase	Alpha-1-antitrypsin
Age	0.004 (*p* = 0.96)	−0.066 (*p* = 0.47)	0.258 (*p* = 0.005)
Time postoperative	0.104 (*p* = 0.25)	−0.072 (*p* = 0.43)	0.021 (*p* = 0.83)
CRP serum level	0.052 (*p* = 0.57)	0.066 (*p* = 0.48)	0.050 (*p* = 0.60)
Current weight	−0.022 (*p* = 0.81)	−0.093 (*p* = 0.31)	−0.051 (*p* = 0.59)
Percentage total weight loss	−0.074 (*p* = 0.42)	0.031 (*p* = 0.74)	−0.123 (*p* = 0.19)
Current BMI	0.046 (*p* = 0.62)	−0.099 (*p* = 0.28)	−0.016 (*p* = 0.86)

Correlations calculated with Spearman’s rho. *p* < 0.005 was considered statistically significant

*CRP* C-reactive protein, *BMI* body mass index

### Elastase

The median elastase level was 443.5 μg/g, ranging from <15 μg/g in three patients to 647 μg/g, significantly lower than the median value of 478 μg/g in the general population (*p* < 0.001). Sixteen patients (13.1 %) had an elastase level below the cutoff of 200 μg/g; in 14 (11.5 %) of these patients, it was below 100 μg/g (Table 2). When patients with a level of <200 μg/g were excluded, the median value was 459 μg/g, still significantly lower than the general population (*p* = 0.02).

Subgroup analyses revealed no differences in median elastase levels between groups (Table 3). No correlation with any of the patient characteristics was found (Table 4), and there was no correlation with the fecal A1AT (rho = 0.101, *p* = 0.28) or calprotectin level (rho = 0.001, *p* = 0.99).

### Alpha-1-Antitrypsin

Alpha-1-antitrypsin was measured in 120 patients. Two cases were excluded because there was not enough material for reliable testing. In five cases, the A1AT level was <0.80 mg/g, but could not be determined more exactly due to insufficient quality of the sample. These cases were not used for determination of the median and reference value. The median A1AT level was 0.51 mg/g, ranging from <0.20 mg/g in 24 patients to 2.20 mg/g (Table 2). This did not differ from the mean level in the general population (*p* = 0.19). Six patients (5.2 %) had an A1AT level higher than the reference value of 1.10 mg/g. Subgroup analysis revealed no significant differences between groups (Table 3). Correlation testing was positive for fecal calprotectin level only, as previously mentioned (Table 4).

After removal of one outlier, a reference value of 1.14 mg/g (90 % confidence interval 1.08–1.27) was determined for A1AT.

## Conclusions

In this study, we found significantly higher levels of fecal calprotectin and lower levels of fecal elastase in patients who underwent RYGB, compared to the general population. The level of fecal alpha-1-antitrypsin was comparable to the general population.

### Calprotectin

The median calprotectin level in our study is 163.5 μg/g, and 87 % of patients had a level above the decision limit of <50 μg/g. We believe this influences the validity of the fecal calprotectin test in the RYGB population. When the current decision limit is maintained for patients who underwent RYGB, a high amount of endoscopies will be performed, which might be unnecessary. It is important that clinicians are aware of this.

There are several possible explanations for the elevation of fecal calprotectin. We hypothesize that the elevated fecal calprotectin levels after RYGB indicate persistent low-grade inflammation or increased epithelial cell turnover in the gastrointestinal tract. This is supported by the positive correlation with alpha-1-antitrypsin [7, 18]. The location and origin of this possible inflammation is unclear. It is also unknown whether surgical alterations of the gastrointestinal anatomy other than RYGB also influence the fecal calprotectin level. RYGB causes hyperplasia of the jejunal mucosa of the Roux-limb, but no macroscopic or histologic evidence of inflammation is found in the majority of patients [14, 22]. An increased inflammatory state can be found in the stomach remnant, and more than half of the patients have chronic gastritis in this remnant [23–25]. However, studies in non-RYGB patients with gastritis found no increase in fecal calprotectin [26]. Another option is that inflammation in the biliopancreatic limb causes the elevated calprotectin. There are no studies describing the macroscopic or histologic appearance of this blind loop. Inflammation in this part of the intestine could be due to the deleterious effect of bile acids and pancreatic enzymes. These are not buffered by ingested food, as would happen in the normal anatomy, and are known to cause damage to the enterocytes [27]. Inflammation might also be caused by bacterial overgrowth in the stomach remnant or the biliopancreatic limb [28]. In non-RYGB patients, small intestinal bacterial overgrowth did not cause an increase in fecal calprotectin [29], but other studies showed that the fecal calprotectin level can change when there are significant changes in gut microbiota [30]. The colon does not seem the most likely location for the increased calprotectin excretion, as its anatomy is not altered in RYGB. However, there are persistent pro-inflammatory changes in the rectum after RYGB [31]. It is possible that the elevation of fecal calprotectin is a sign of low-grade colonic inflammation, in line with these findings.

We feel that methodological errors are unlikely. Calprotectin was collected and stored according to current standards. It is known to remain stable for several days at room temperature and longer when frozen [7]. All fecal tests were done in one batch by the same experienced laboratory. All patients had undergone surgery at least 1 year prior to inclusion and had no severe gastrointestinal complaints. Strict exclusion criteria were set for major factors that influence calprotectin levels, but not for factors such as age, fiber intake, and physical activity that are known to have only minimal influence [32, 33]. PPI and NSAID use can influence calprotectin; therefore, these drugs were discontinued for at least 3 days prior to feces collection. Subgroup analysis of patients who discontinued the PPI for 3–7 days versus those who had stopped PPI use for more than 7 days before collection revealed no significant difference in fecal calprotectin levels. Only five patients occasionally used NSAIDs prior to feces collection. Calprotectin level in these patients did not differ from patients who never used NSAIDs. Furthermore, calprotectin levels did not correlate with the current BMI, percentage total weight loss, or age in this study.

The determination of a new decision limit for calprotectin after RYGB is not possible based on this study. A large cohort of RYGB patients diagnosed postoperatively with IBD would be needed for this. In practice, when the prevalence of IBD is taken into account, this is not a feasible study design.

### Elastase

The median fecal elastase level is lower in patients after RYGB than in the general population, as was also found in a previous study [6]. However, the majority of patients had a level above the limit for pancreatic insufficiency. None of the 16 patients (13 %) with a fecal elastase level below the reference value had clinical or biochemical evidence of pancreatic insufficiency. Therefore, it is possible that the specificity after RYGB is lower than the known specificity of 90–93 % in the general population [10]. This can only be confirmed by performing invasive pancreatic function tests in these patients, which was not done in the course of this study.

There was evidently no correlation between fecal calprotectin and elastase in this study, contrary to previous findings [6, 34]. This may be explained by a smaller study group and the selection of patients with pancreatic diseases in these previous studies.

### Alpha-1-Antitrypsin

The reference value and the median in the study population did not differ significantly from the current reference value of 1.10 mg/L and the median in the general population. We thus conclude that the reliability of the fecal A1AT test is not impaired after RYGB.

### Limitations

This study has several limitations. No comparison is possible with preoperative fecal values of the participants due to the cross-sectional design. However, we deem it unlikely that all patients already had elevated calprotectin levels before surgery as these patients had no gastrointestinal disease prior to RYGB. Previous studies concerning calprotectin levels in obese patients never showed levels as high as observed in this study [17, 33]. Second, no endoscopy, invasive pancreatic function tests, or radiological examinations were performed in this study. These tests might have clarified some of the questions this study raises and should certainly be included in future studies. Strict inclusion and exclusion criteria were set to prevent confounding. This means that the results cannot be transferred directly to the whole RYGB population at whichever period postoperative. Despite the limitations, we believe the results of this study are apparent enough to question the validity of the current decision limit for fecal calprotectin, and to a lesser extent elastase, in patients who underwent RYGB.
